# Integrating an Elder Mistreatment Intervention Protocol Into the Emergency Department Electronic Health Record

**DOI:** 10.1093/geroni/igaf122.089

**Published:** 2025-12-31

**Authors:** Jason Burnett, Charles Maddow, Michael Cannell

**Affiliations:** UTHealth Houston, Houston, Texas, United States; UTHealth Houston, Houston, Texas, United States; TCU, Fort Worth, Texas, United States

## Abstract

Experiencing mistreatment in later life significantly increases the risk of morbidity and mortality. These heightened risks are often preceded by or closely linked to emergency department visits and subsequent hospitalizations, which are more frequent among abused older adults compared to those who have not experienced mistreatment. Despite this, standardized screening for elder mistreatment (EM) remains low across clinical settings, including emergency departments—where trauma and violence-related injuries are routinely assessed and treated. As a result, emergency departments present a crucial opportunity for improving the detection of elder mistreatment and providing potentially life-saving interventions. However, a key barrier to screening in these settings is the absence of integrated EM screening and response protocols within electronic health records, which guide most routine assessments in emergency care. This presentation outlines the process of implementing an evidence-informed EM screening and response protocol in a large Level III trauma center. Findings highlight the shift in EM screening rates among adults aged 65 and older admitted to the emergency department—from approximately 2% before implementation to 85% afterward. Additionally, challenges, barriers, and strategies for successful implementation are explored.

